# Supplementary material for: Actin filaments accumulated in the nucleus remain in the vicinity of condensing chromosomes in the zebrafish early embryo
Source: Biol Open. 2023 May 17;12(5):bio059783. doi: 10.1242/bio.059783 (PMC10214854; doi:10.1242/bio.059783)
Supplement: Supplementary information [file biolopen-12-059783-s1.pdf]

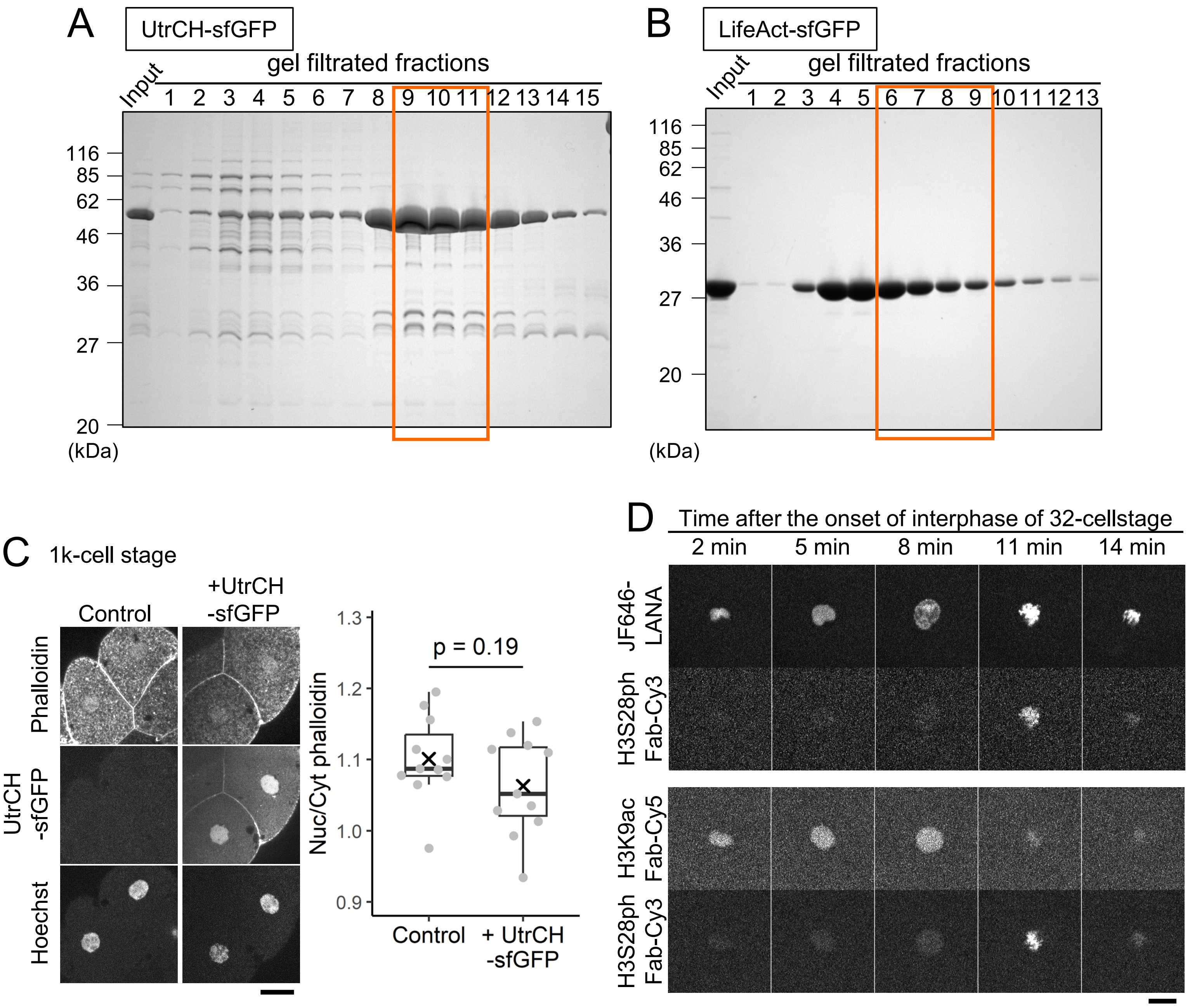

Fig. S1. Actin and chromatin probes.

(**A** and **B**) Purification of actin probes. UtrCH-sfGFP and LifeAct-sfGFP were expressed in *E. coli* and purified through GST-sepharose followed by GST removal and gel filtration. Fractions though a gel filtration column and inputs were separated on polyacrylamide gels and stained with Coomassie Blue. The positions of size standards are indicated on the left. The fractions #9-11 of UtrCH-sfGFP (**A**) and #6-9 of LifeAct-sfGFP (**B**), as indicated by orange boxes, through gel filtration were pooled and used as actin probes for live imaging. (**C**) Zebrafish embryos were injected with UtrCH-sfGFP or PBS as the vehicle. Embryos were fixed at the 1k-cell stage, and stained with Acti-stain™ 555 phalloidin and Hoechst. Single confocal sections are shown on the left. N/C intensity ratio of phalloidin in nuclei with condensing chromosomes (N = 11 cells from three embryos) are shown on the right. In the box plots, center lines show the medians; box limits indicate the 25th and 75th percentiles; whiskers extend 1.5 times the interquartile range from the 25th to 75th percentiles; × indicates the means; and data points are plotted as gray dots. *p*-values obtained with a Student's *t*-test (unpaired, two-tailed) is also shown. UtrCH-sfGFP injection did not increase the nuclear phalloidin level. (**D**) JF646-LANA labels mitotic chromosomes as well as interphase chromatin. Zebrafish embryos were injected with either JF646-LANA or H3K9ac Fab-Cy5 as a chromatin marker, together with H3S28ph Fab-Cy3 as a mitotic chromosome marker. Confocal sections were acquired every 1 min. Single confocal sections are shown for JF646-LANA or H3K9ac Fab-Cy5, and H3S28ph Fab-Cy3 at the 32-cell stage. JF646-LANA clearly highlights chromatin throughout the cell cycle, with intense signals on condensed chromosomes from prophase (8 min) to prometaphase (11 min) and anaphase (14 min), compared to H3K9ac Fab-Cy5 signals that become weaker during mitosis (11 and 14 min). Scale bars: 100 µm (**C**), 20 µm (**D**).

Biology Open • Supplementary information

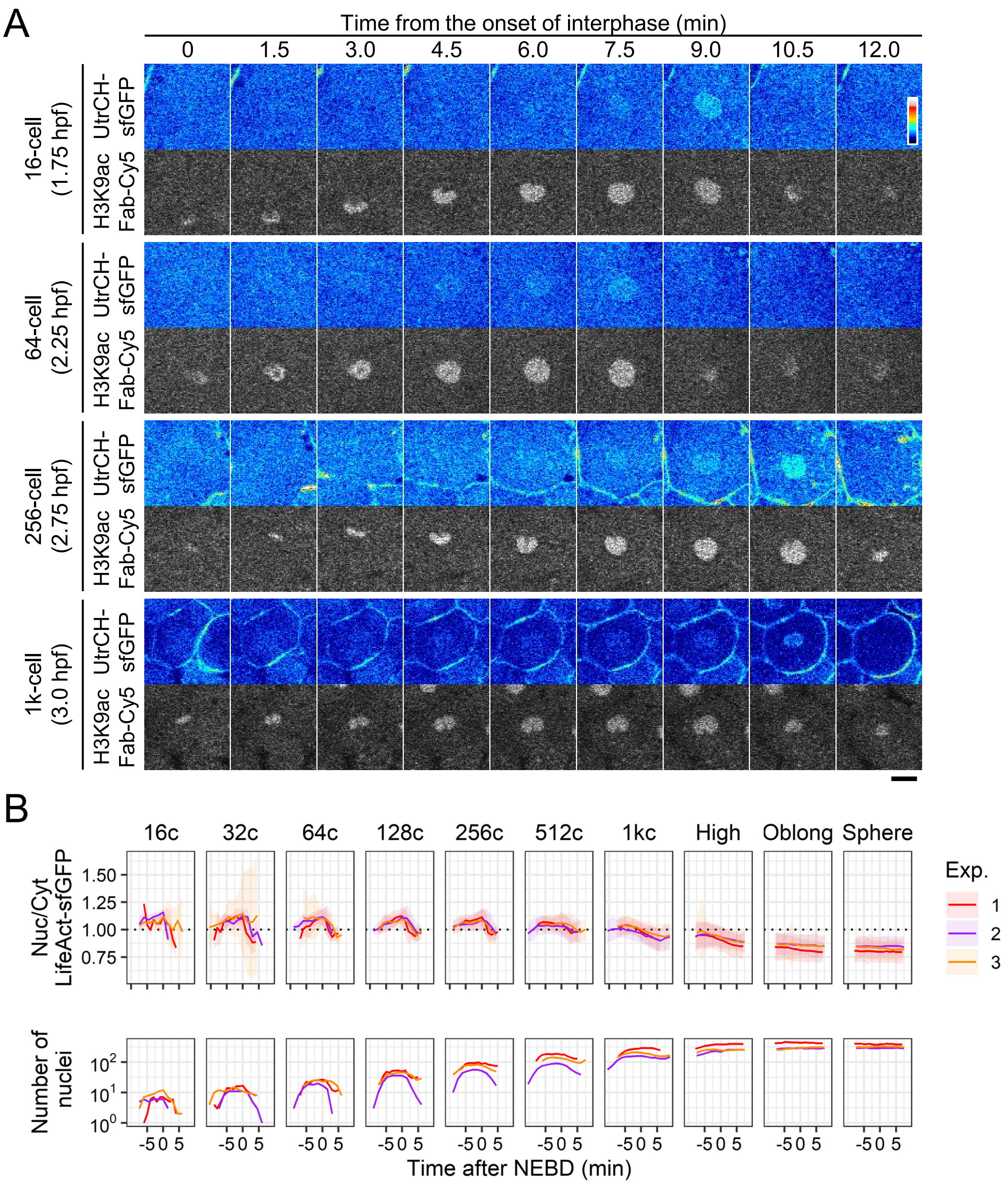

Fig. S2. Actin accumulated in the interphase nuclei at the 16-, 64-, 256-, and 1k-cell stages. Zebrafish embryos were injected with UtrCH-sfGFP and H3K9ac Fab-Cy5, and images were acquired using the same procedure as described in Figure 1D and 2A. (**A**) Single confocal sections of a nucleus at the 16-, 64-, 256-, 1k-cell stages are depicted with the time (min) after the onset of the interphase. Scale bar: 20 µm. (**B**) The N/C intensity ratios of LifeAct-sfGFP were measured from three independent experiments. One embryo was analyzed in each experiment. Mean values of N/C ratios are plotted with standard deviations (s. d.). N/C ratios of LifeAct-sfGFP are above 1.0 (dashed line) up to the 1k-cell stage.

Biology Open • Supplementary information

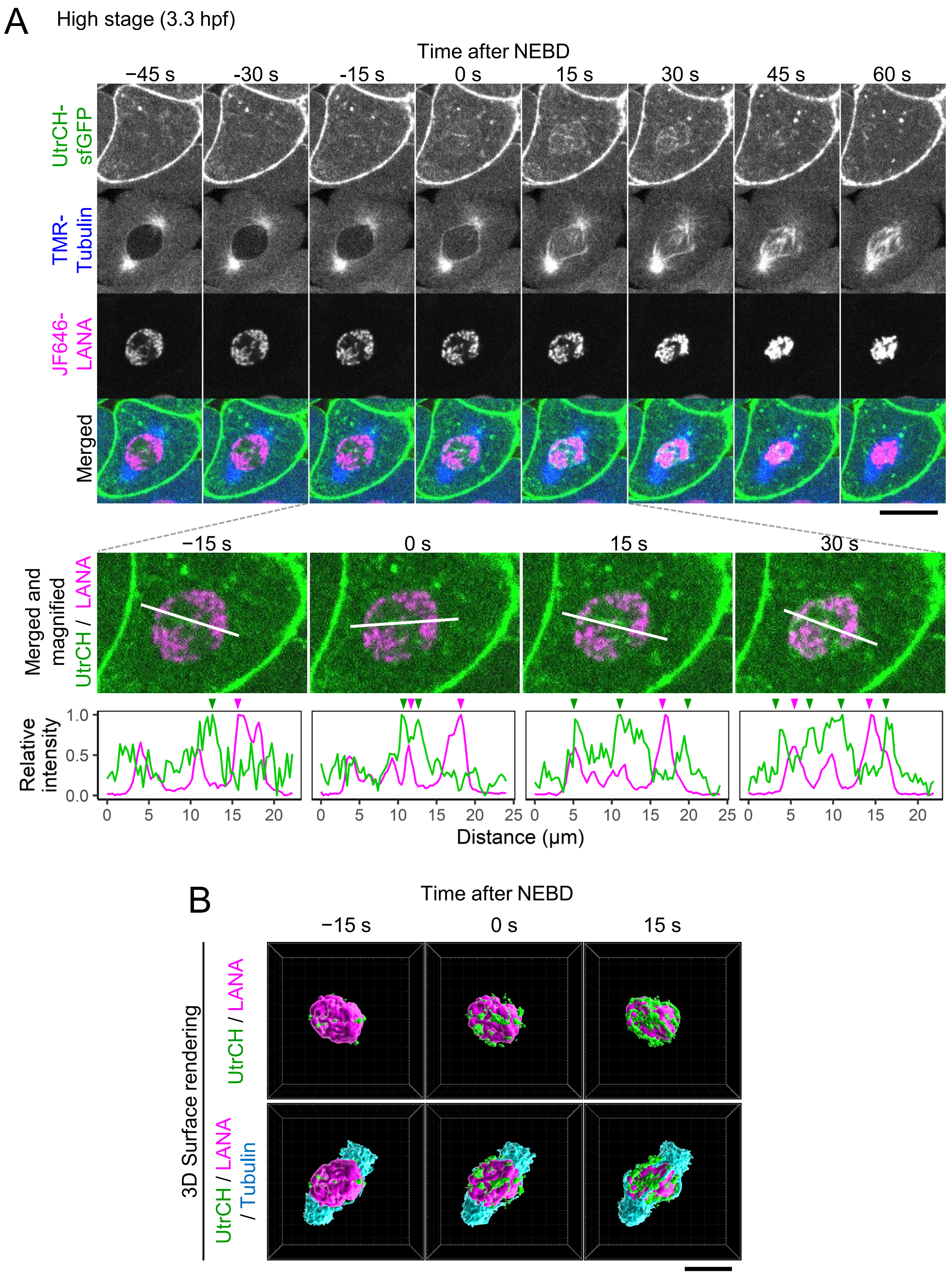

Fig. S3. Actin patches partially remained in the vicinity of chromosomes during prometaphase and disappeared at the metaphase in the high stage embryos.

Zebrafish embryos were injected with UtrCH-sfGFP, TMR-tubulin, and JF646-LANA. Every 15 s, confocal sections were acquired. (**A**) Single sections of UtrCH-sfGFP, TMR-tubulin, and JF646-LANA at the high stage embryo, and their merged and magnified images are shown. Relative intensity profile plots of lines are indicated at the bottom. The intensity is normalized using maximum and minimum intensity. UtrCH-sfGFP signals are located in the vicinity of chromosomes. (**B**) The surface rendering three-dimensional images are shown (green: UtrCH-sfGFP, magenta: JF646-LANA, cyan: TMR-Tubulin). UtrCH-sfGFP signals appeared around condensing chromosomes after NEBD (from 0 s to 45 s) and disappeared before the chromosomes were aligned at the metaphase (60 s). Just after NEBD (0 s and 15 s), TMR-Tubulin does not appear to reach chromosomes associated with UtrCH-sfGFP patches. TMR-Tubulin appears to capture condensing chromosomes (0 s to 30 s). See also Movie 6. Scale bars: 20 μm.

Biology Open • Supplementary information

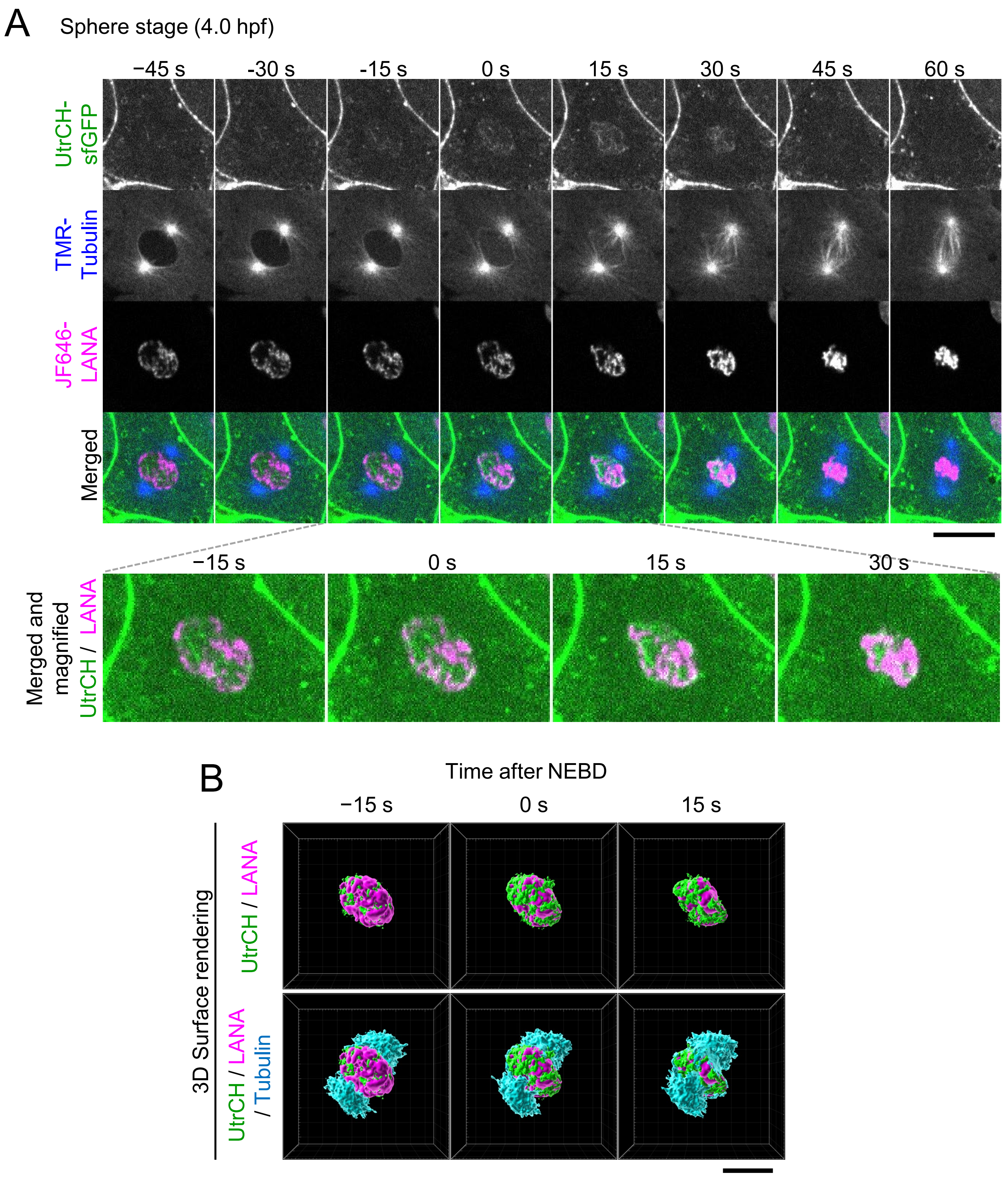

Fig. S4. Actin patches appeared during prophase to prometaphase and disappeared at the metaphase in the sphere stage embryo.

Zebrafish embryos were injected with UtrCH-sfGFP, TMR-labeled tubulin, and JF646-LANA. Every 15 s, confocal sections were acquired. (**A**) Single sections for UtrCH-sfGFP, TMR-labeled tubulin, and JF646-LANA in sphere stage embryo, and their merged and magnified images are shown. (**B**) The surface rendering three-dimensional images are shown (green: UtrCH-sfGFP, magenta: JF646-LANA, cyan: TMR-Tubulin). Weak UtrCH-sfGFP signals were observed in the vicinity of chromosomes after NEBD (0 s and 15 s) and disappeared (45 s) before chromosomes aligned at the metaphase (60 s). Scale bars: 20 µm.

Biology Open • Supplementary information

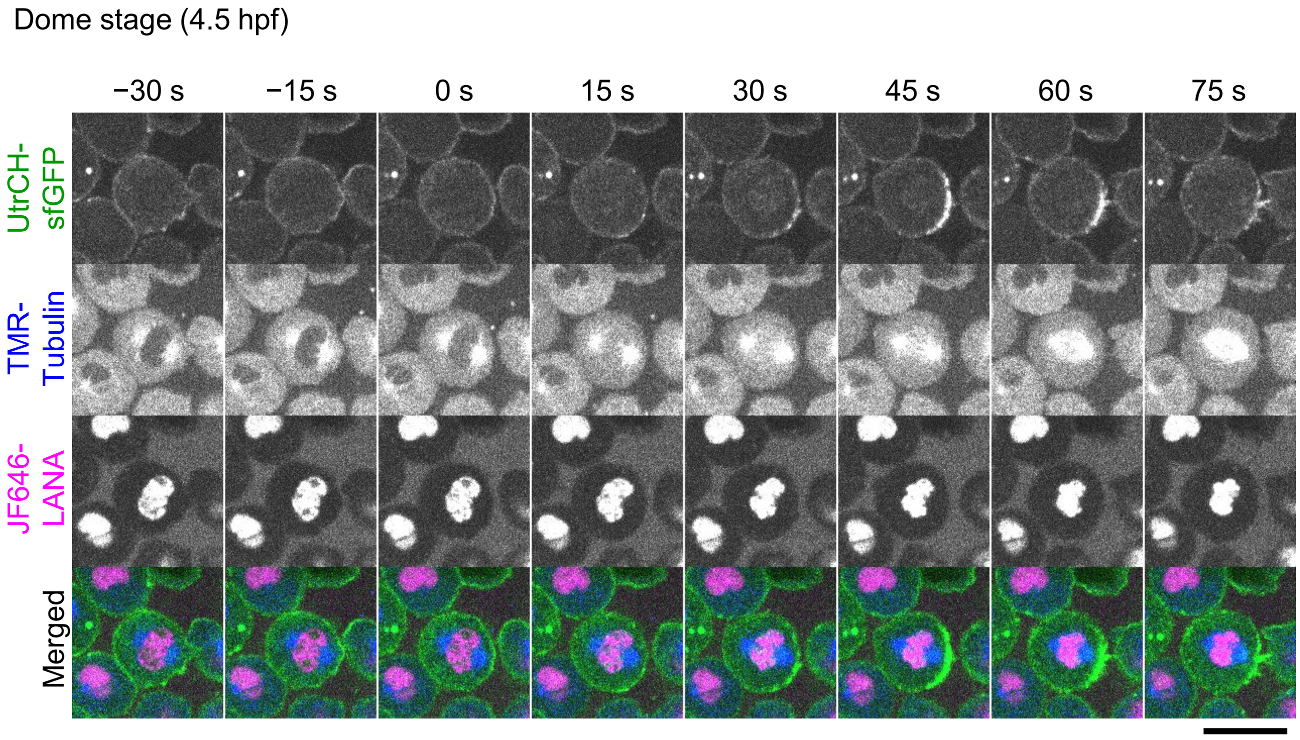

Fig. S5. Weak actin patches in the dome stage embryo.

Zebrafish embryos were injected with UtrCH-sfGFP, TMR-labeled tubulin, and JF646-LANA. Every 15 s, confocal sections were acquired. Single sections for UtrCH-sfGFP, TMR-tubulin, and JF646-LANA in the dome stage embryo, and their merged images are shown. At this stage, very weak UtrCH-sfGFP signals appeared during prometaphase (from 15 s to 45 s). Scale bar; 20 µm.

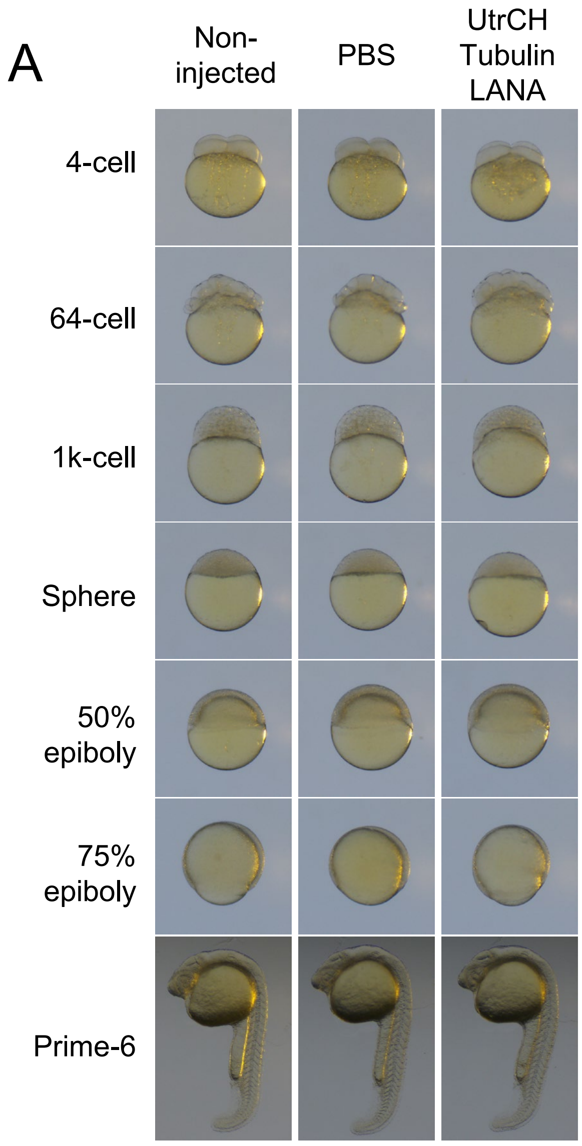

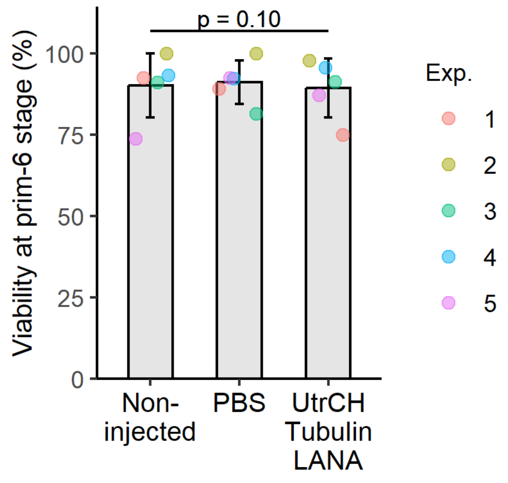

Viability at prime-6 stage %

Exp.	Non-injected	PBS	UtrCH Tubulin LANA
1	92.6 (54)	89.1 (46)	75.0 (52)
2	100 (42)	100 (47)	97.8 (46)
3	91.2 (68)	81.5 (54)	91.4 (58)
4	93.3 (60)	92.4 (66)	95.7 (69)
5	73.8 (61)	92.5 (67)	87.3 (55)

(number of embryo)

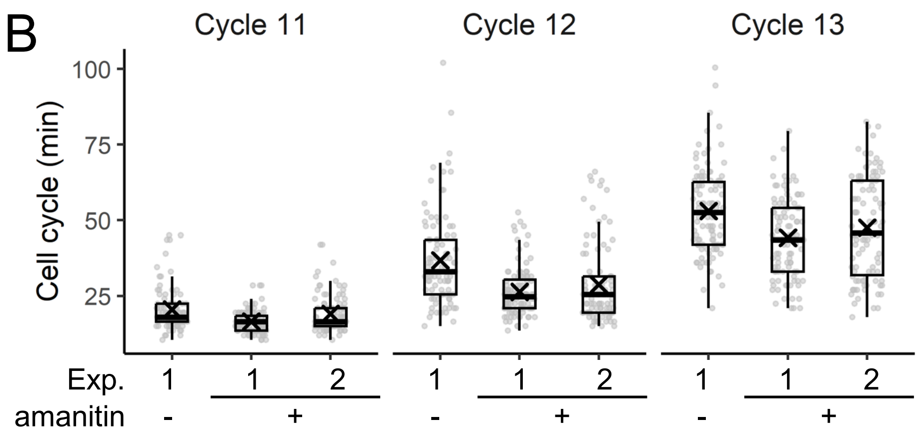

Fig. S6. Embryo viability after the injection of probes and cell cycle length in a transcription inhibitor.

(**A**) Viability of embryos injected with probes. Embryos were injected with a mixture of UtrCH-sfGFP, TMR-labeled Tubulin, and JF646-LANA, or with PBS as the vehicle, and their development up to the prime-6 stage was analyzed. Five independent experiments were performed. Representative images of embryos at each stage, acquired using a stereo microscope (Nikon, SMZ 745T), and the embryo viability at the prime-6 stage are shown on the left and right, respectively. One-way factorial ANOVA was used to obtain the *p*-value. (**B**) Effect of α-amanitin on the cell cycle. Embryos were injected with α-amanitin or PBS, and then with UtrCH-sfGFP and H3K9ac Fab-Cy5. Every 90 s, 25 z-stack fluorescence images with 4 µm intervals were acquired. The cell cycle length of 100 cells at the 1k-cell stage (cell division cycle 11) and later (cycles 12 and 13) were measured. In the box plots, center lines show the medians; box limits indicate the 25th and 75th percentiles; whiskers extend 1.5 times the interquartile range from the 25th to 75th percentiles; × indicates the mean; and data points are plotted as gray dots. Embryos injected with α-amanitin showed slightly shorter cell cycle at cycles 12 and 13, which might be caused by the inhibition of zygotic genome activation.

Biology Open • Supplementary information

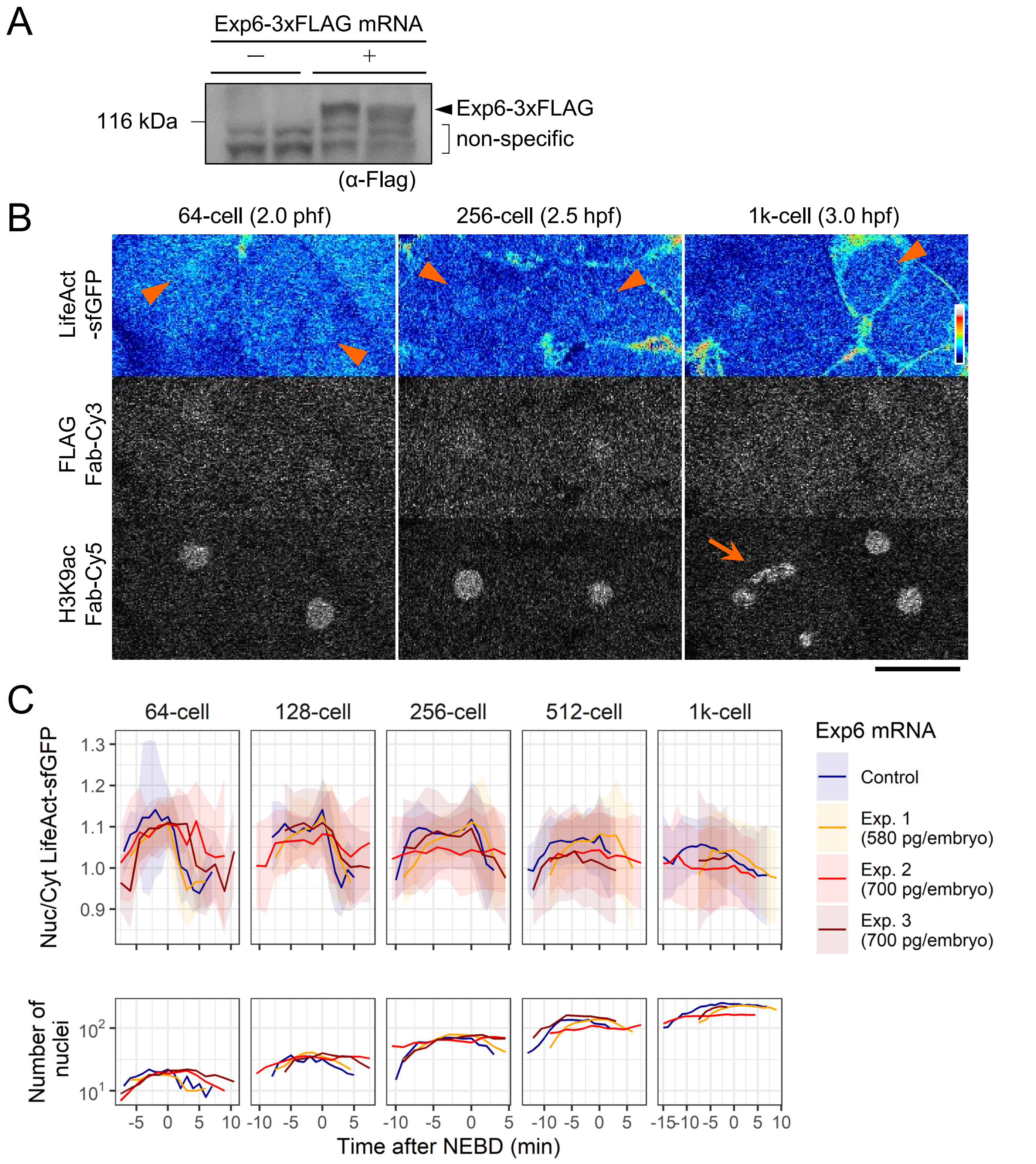

Fig. S7. Expotin 6 expression did not decrease nuclear actin levels but caused severe defects in cytokinesis.

(**A**) To express zebrafish exportin 6 (Exp6), embryos were injected with mRNA of 3×FLAG-tagged Exp6, and were allowed to develop for 5.3 h (up to 50% epiboly). After de-yolking, embryos were washed and dissolved in SDS-gel loading buffer. Two lysates from 10 embryos each for control (−) and mRNA-injected (+) were separated on a SDS-polyacrylamide gel for western blotting with anti-FLAG antibody. The positions of the size standard and Exp6-3×FLAG are indicated on the left and right, respectively. (**B**) Zebrafish embryos were injected with mRNA encoding Exp6-3×FLAG, LifeAct-sfGFP, Cy3-labeled anti-FLAG Fab, and Cy5-labeled anti-H3K9ac Fab. Single confocal sections at the 64-, 256-, and 1k-cell stages are shown. LifeAct-sfGFP and FLAG Fab-Cy3 are slightly concentrated in nuclei (arrowheads). Cytokinesis defects were induced and resulted in binucleation (arrow). Scale bar: 50 µm. (**C**) Three independent mRNA injection experiments and one control were performed as in (**B**). The mean values of N/C ratios of LifeAct-sfGFP were plotted with s. d. and the number of nuclei. N/C ratio of LifeAct-sfGFP was not decreased by Exp6-3×FLAG expression.

Biology Open • Supplementary information

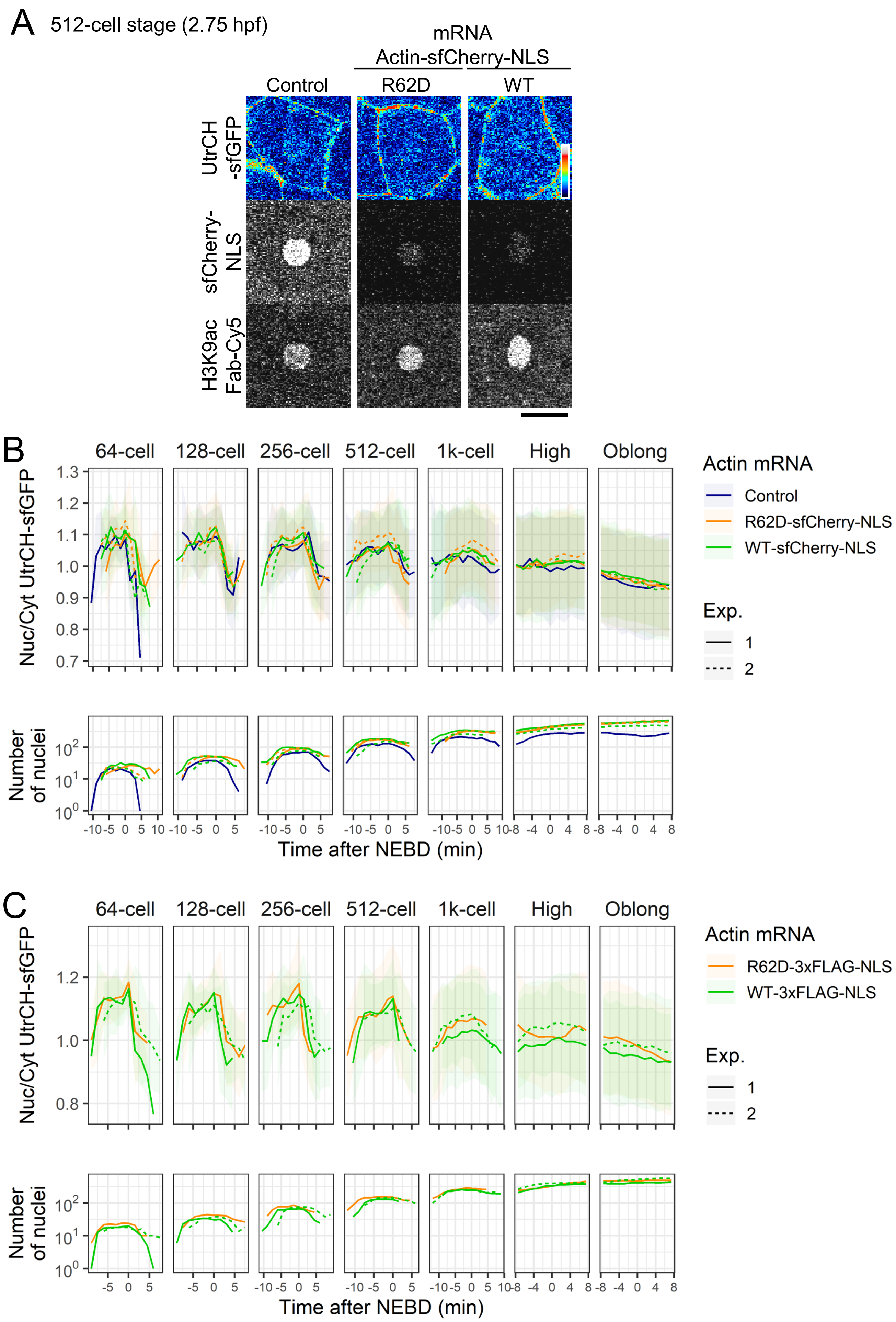

Biology Open • Supplementary information

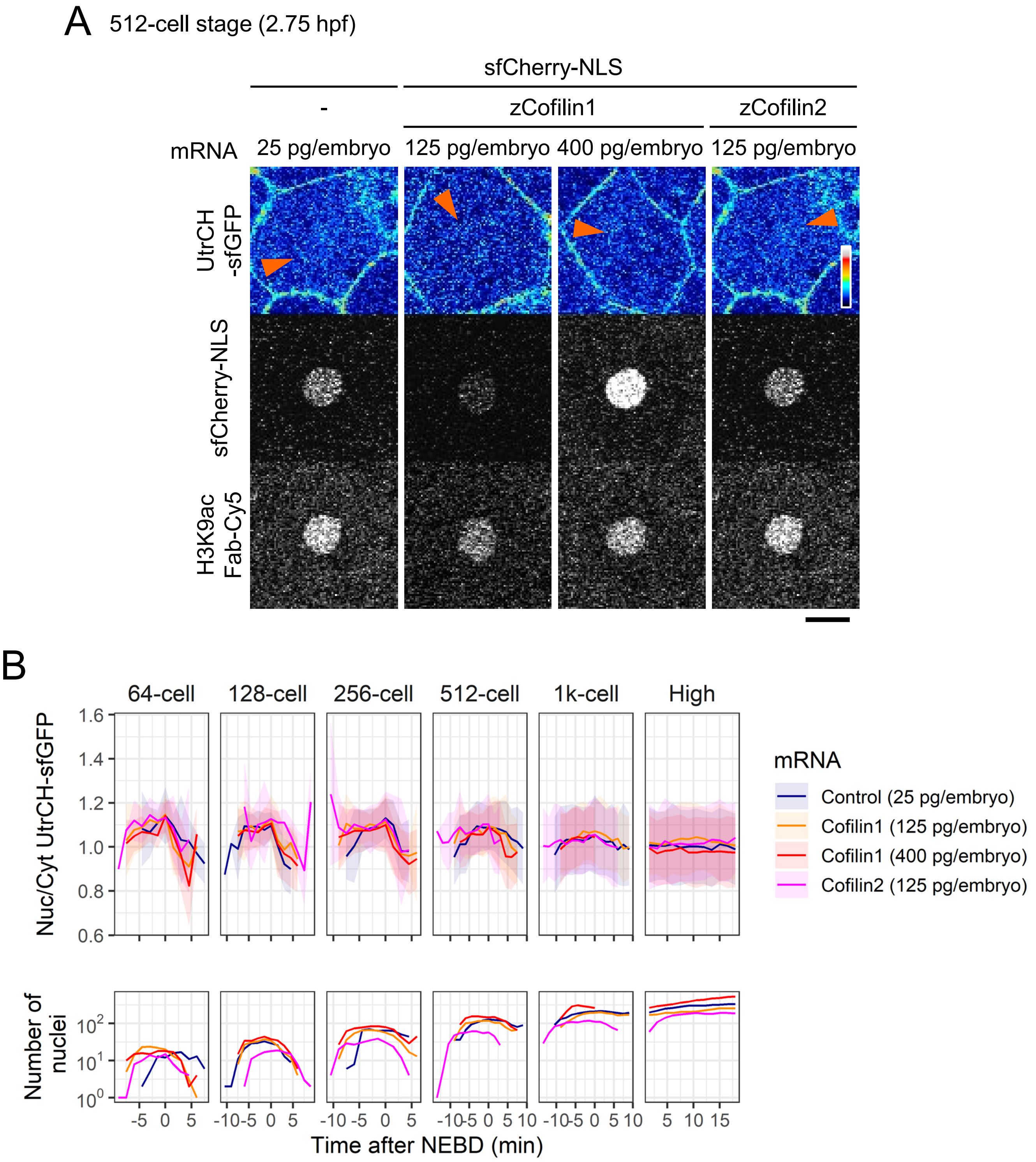

Fig. S9. Expression of NLS-tagged cofilin1 did not decrease nuclear F-actin levels. Actin depolymerizing factors, zebrafish cofilin1 and cofilin2, each tagged with sfCherry-NLS, were expressed by mRNA injection at 1-cell stage embryos. sfCherry-NLS was used as the control. UtrCH-sfGFP and Cy5-H3K9ac Fab were co-injected and 25-stack confocal images with 4 μm intervals were acquired every 90 s. (A) Single confocal sections at the 512-cell stage are shown. Although cofilin1-sfCherry-NLS was accumulated in the nucleus, nuclear UtrCH-sfGFP signals were still detected (arrowheads). Scale bar: 20 μm. (B) Mean values of N/C intensity of UtrCH-sfGFP are plotted with the s. d. and the number of nuclei. No substantial difference was observed.

Biology Open • Supplementary information

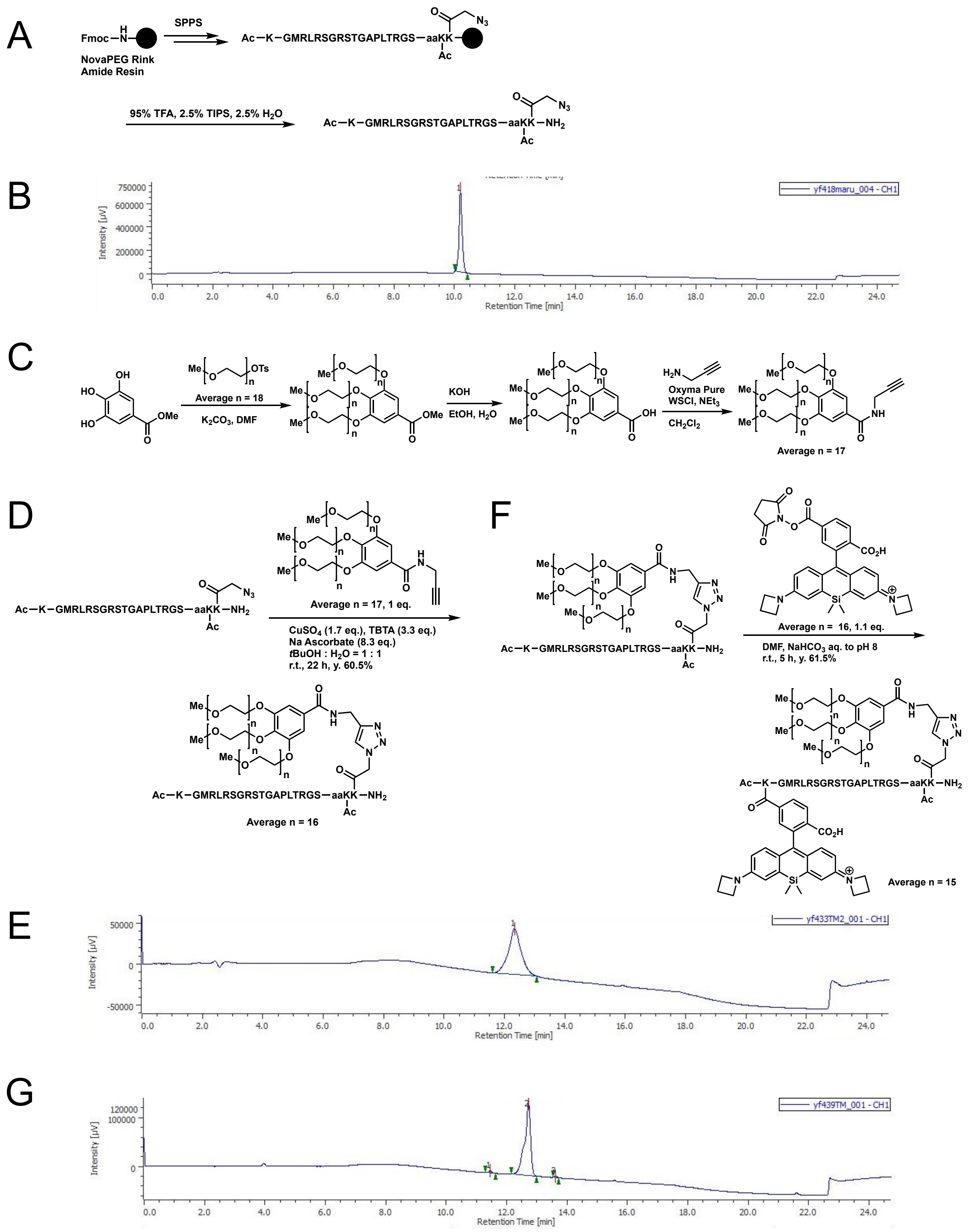

Fig. S10. Synthesis of PEG_{750}-LANA-JF646 (JF646-LANA).

The reaction schemes for PEG_{750}-LANA-JF646 synthesis (**A**, **C**, **D**, and **F**) and analytical HPLC chromatograms (**B**, **E**, and **G**) are shown. (**A**) Synthesis of LANA peptide by sequential addition of Fmoc-amino acid on a NovaPEG-Rink-amide-resin. (**C**) Synthesis of 3,4,5-Tris(PEG_{750})-N-(prop-2-yn- 1-yl)benzamide. (**D**) Synthesis of PEG_{750}-LANA. (**F**) Synthesis of PEG_{750}-LANA-JF646. (**B**, **E**, and **G**) Products were analyzed by analytical HPLC using a YMC-Triart Phenyl C18 (4.6 mm I.D. x 150 mm) column with a linear gradient of 2%–90% acetonitrile in 0.1% aqueous TFA over 3–15 min. (**B**) LANA peptide (Retention time: 10.2 min, 22.9 mg, yield 16.9%). (**E**) PEG_{750}-LANA (Retention time: 12.3 min, 2.51 mg, 0.436 µmol, yield 60.5%, yellow powder). (**G**) PEG_{750}-LANA-JF646 (Retention time: 12.7 min, 1.56 mg, 0.246 µmol, yield 61.5%, powder).

Biology Open • Supplementary information

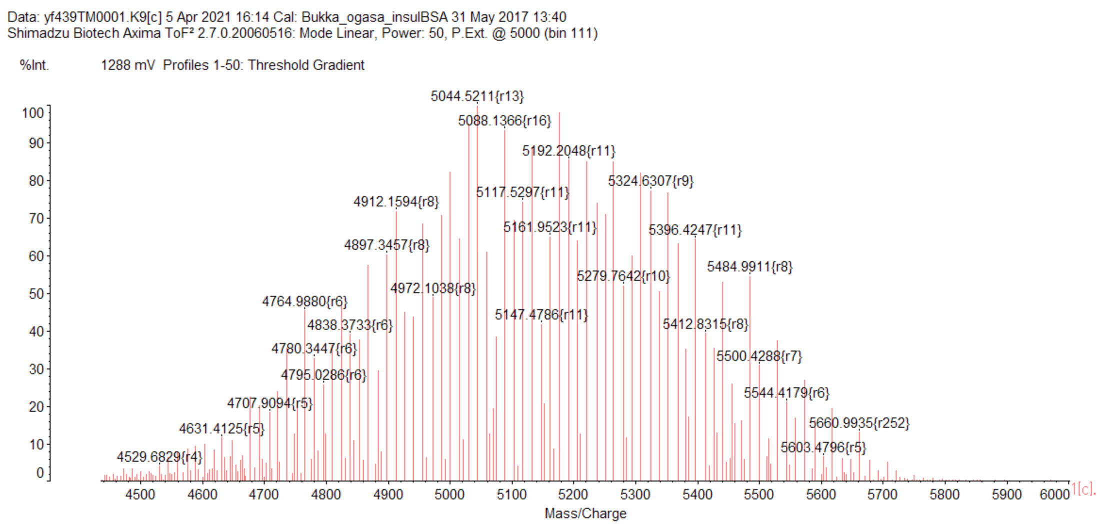

Zoomed view

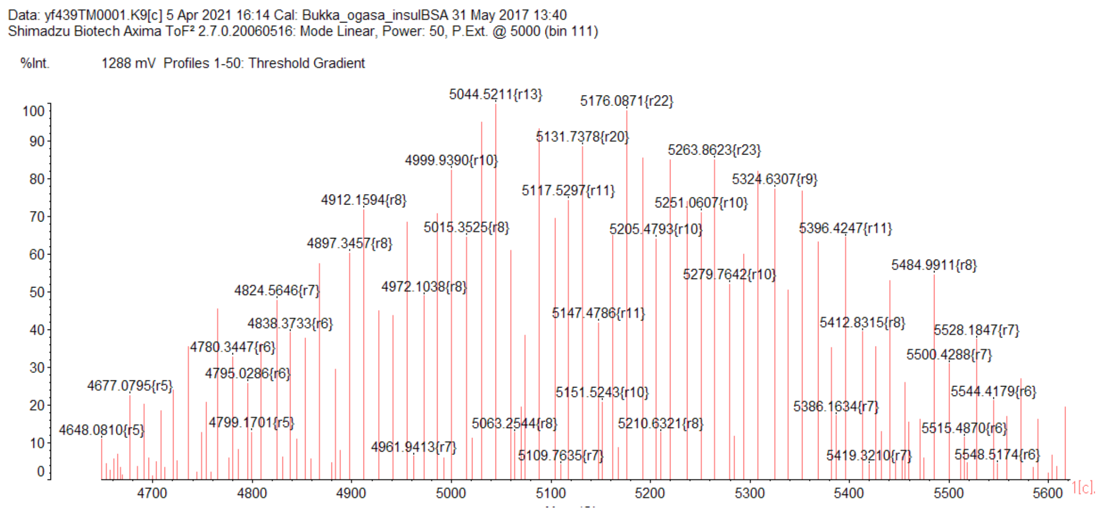

Fig. S11. Mass spectrometry analysis of JF646-LANA.
Wide (**A**) and zoomed (**B**) views of the MALDI-TOFMS spectrum of PEG_{750}-LANA-JF646 are shown. One of the peaks of the highest intensity has *m*/*z* of 5263.9, which corresponds to ethylene glycol n = 15 (Calculated: 5260.9 $[M]^+$ $C_{236}H_{408}N_{45}O_{83}SSi^+$).

Biology Open • Supplementary information

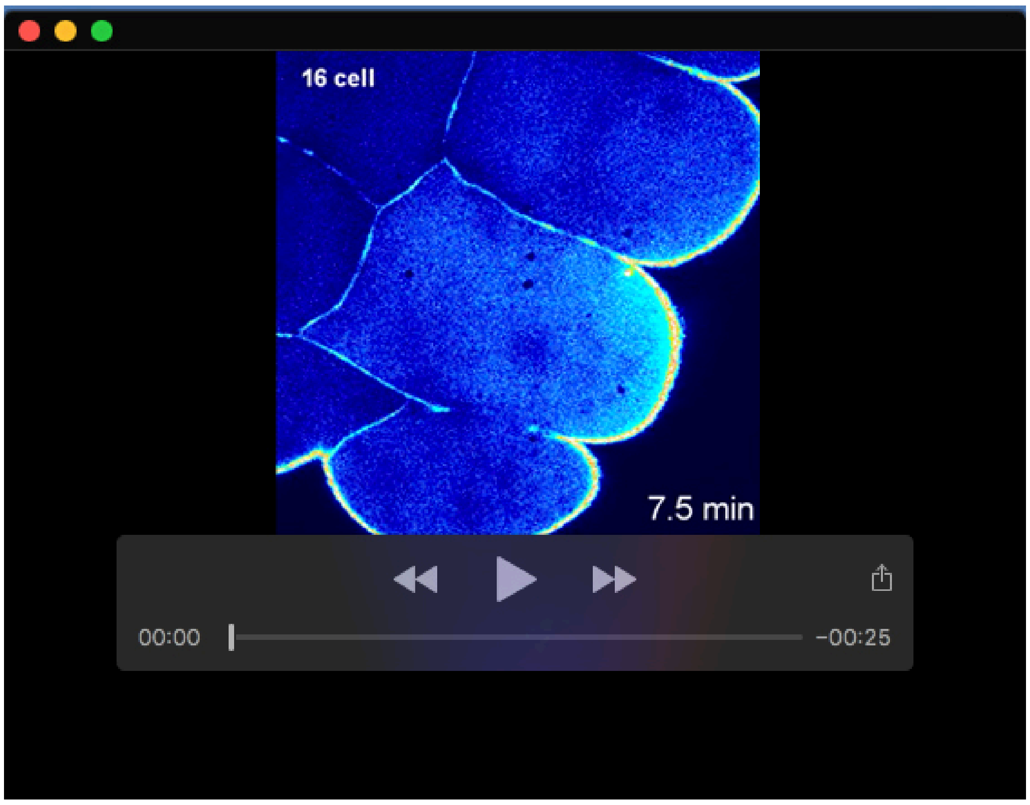

Movie 1. F-actin dynamics visualized using UtrCH-sfGFP in the zebrafish embryo from the 8-cell stage to the high stage.
Zebrafish embryos were injected with UtrCH-sfGFP and H3K9ac Fab-Cy5. Every 90 s, fluorescence images of the 8-cell stage to the high stage were acquired using a confocal microscope. Single confocal sections of UtrCH-sfGFP signals from the 8-cell stage (1.25 hpf) to high stage (3.5 hpf) are shown.

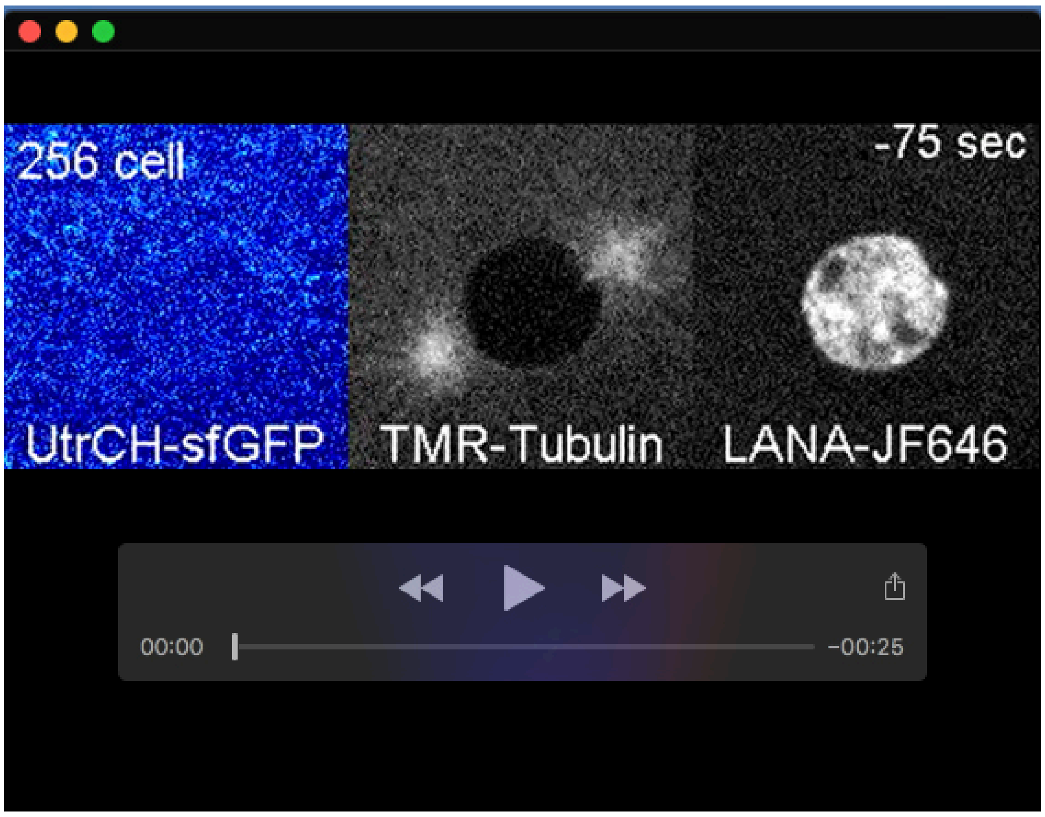

Movie 2. F-actin dynamics during chromosome condensation at the 256-cell stage. Zebrafish embryos were injected with UtrCH-sfGFP, TMR-labeled tubulin, and JF646-LANA. Every 15 s, fluorescence images of the 256-cell stage (2.5 hpf) were acquired using a confocal microscope. UtrCH-sfGFP patches located in the vicinity of chromosomes from NEBD (0 s) to prometaphase (30 s) and disappeared before metaphase (60s). Time after NEBD is indicated.

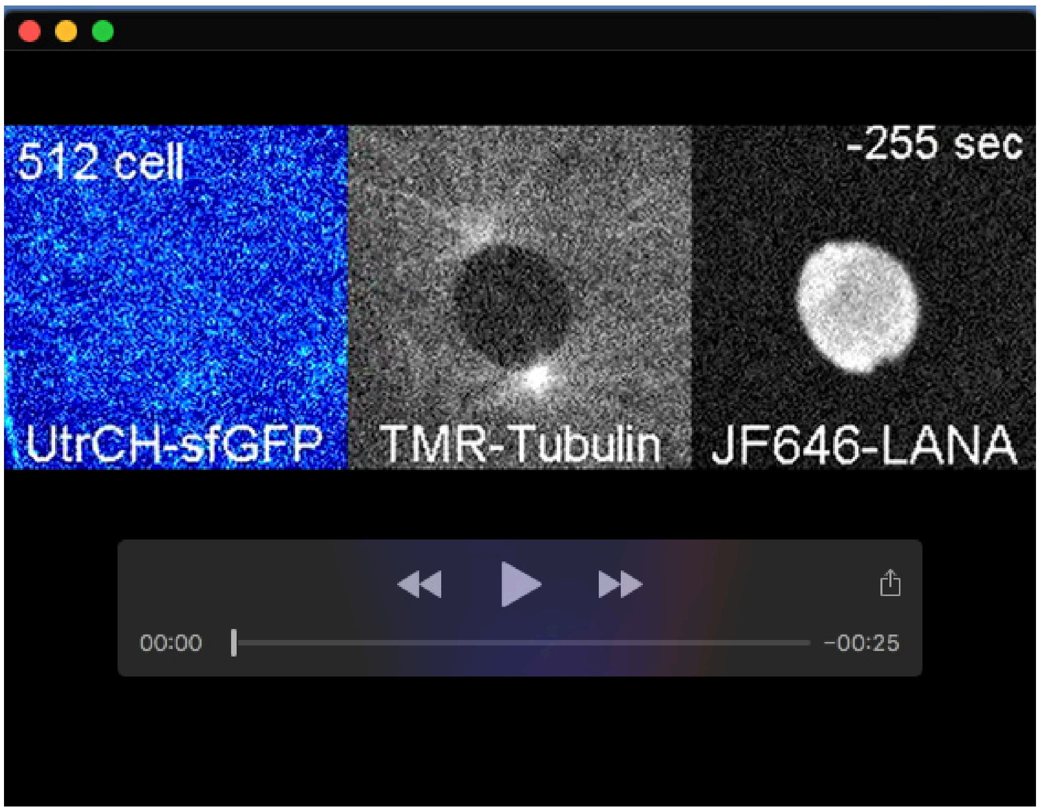

Movie 3. F-actin dynamics during chromosome condensation at the 512-cell stage. Zebrafish embryos were injected with UtrCH-sfGFP, TMR-Tubulin, and JF646-LANA. Every 15 s, fluorescence images of the 512-cell stage were acquired using a confocal microscope. UtrCH-sfGFP patches located in the vicinity of chromosomes from prophase (−30 s) to prometaphase (30 s) and disappeared before metaphase (60 s). Time after NEBD is indicated.

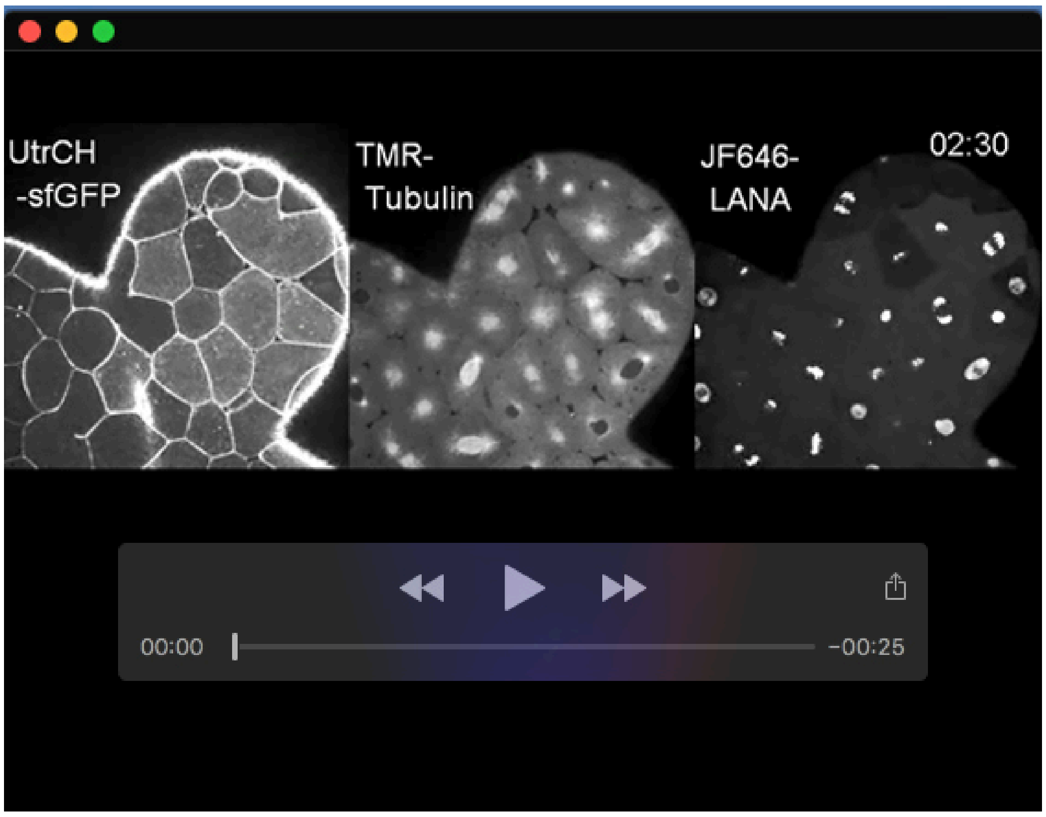

Movie 4. Low power view of F-actin dynamics during chromosome condensation at the 1k-cell stage. Zebrafish embryos were injected with UtrCH-sfGFP, TMR-Tubulin, and JF646-LANA. Every 15 s, fluorescence images of the 1k-cell stage were acquired using a confocal microscope. A low power view is shown to cover many cells. Elapse time from the onset of imaging is shown on top right. UtrCH-sfGFP accumulated in the nucleus and remained in the vicinity of chromosomes.

Biology Open • Supplementary information

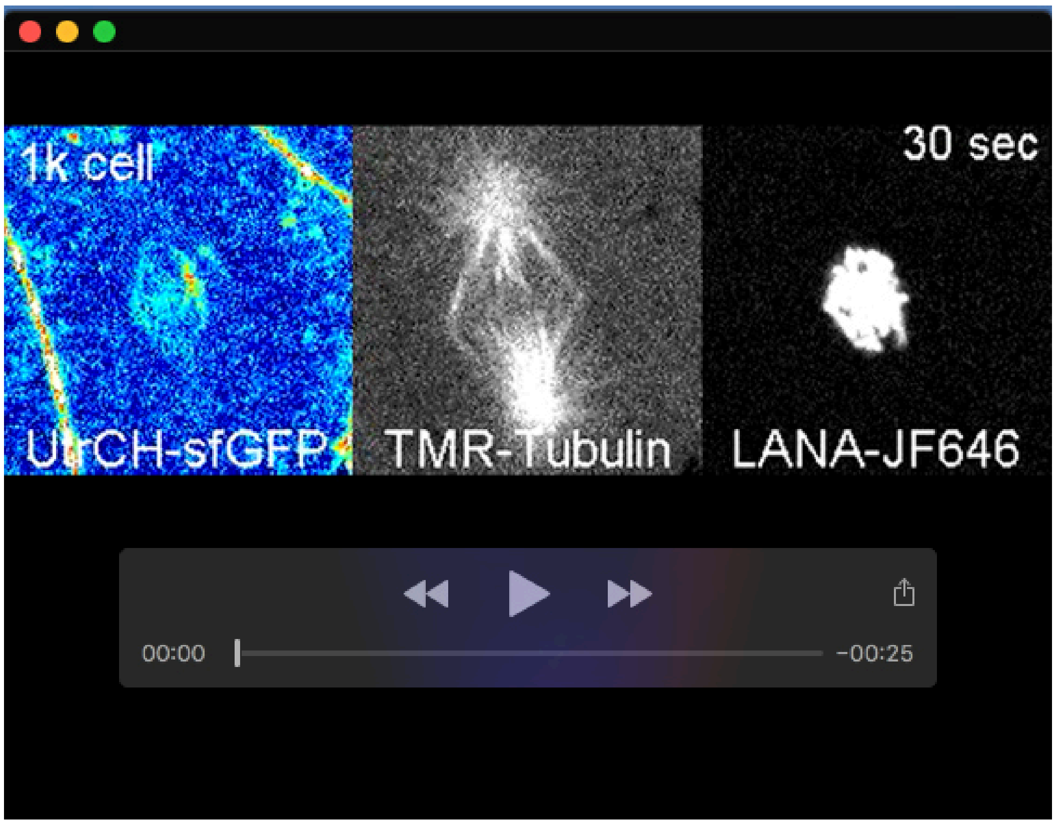

Movie 5. F-actin dynamics during chromosome condensation at the 1k-cell stage. A magnified view of a representative nucleus at the 1k-cell stage. UtrCH-sfGFP accumulated in the nucleus (−15 s) and remained in the vicinity of chromosomes during prometaphase (0 s to 30 s) and disappeared before metaphase (60 s). Time after NEBD is indicated.

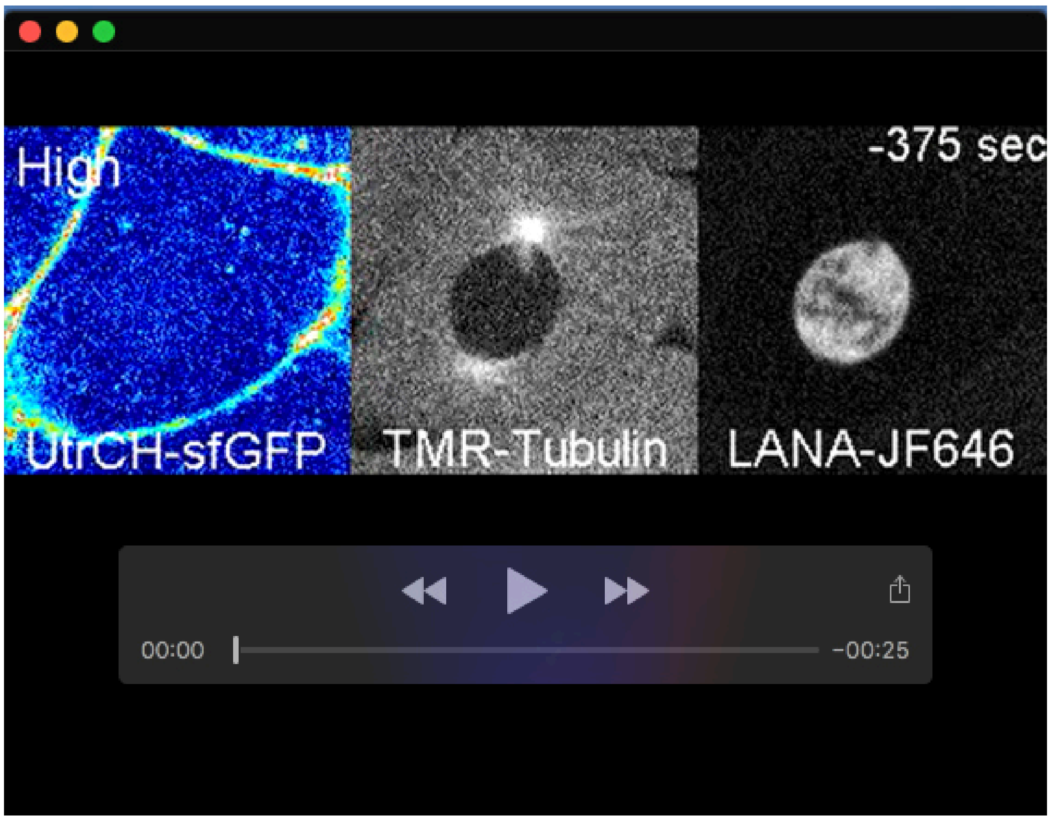

Movie 6. F-actin dynamics during chromosome condensation at the high stage. Zebrafish embryos were injected with UtrCH-sfGFP, TMR-Tubulin, and JF646-LANA. Every 15 s, fluorescence images of the high stage (3.3 hpf) were acquired using a confocal microscope. UtrCH-sfGFP patches located in the vicinity of chromosomes at NEBD (0 s) and remained during the prometaphase and disappeared before metaphase (60 s). Time after NEBD is indicated.

Biology Open • Supplementary information
